# A Meta-Analysis Strategy for Gene Prioritization Using Gene Expression, SNP Genotype, and eQTL Data

**DOI:** 10.1155/2015/576349

**Published:** 2015-03-22

**Authors:** Jingmin Che, Miyoung Shin

**Affiliations:** Bio-Intelligence & Data Mining Lab, School of Electronics Engineering, Kyungpook National University, 1370 Sankyuk-dong, Buk-gu, Daegu 702-701, Republic of Korea

## Abstract

In order to understand disease pathogenesis, improve medical diagnosis, or discover effective drug targets, it is important to identify significant genes deeply involved in human disease. For this purpose, many earlier approaches attempted to prioritize candidate genes using gene expression profiles or SNP genotype data, but they often suffer from producing many false-positive results. To address this issue, in this paper, we propose a meta-analysis strategy for gene prioritization that employs three different genetic resources—gene expression data, single nucleotide polymorphism (SNP) genotype data, and expression quantitative trait loci (eQTL) data—in an integrative manner. For integration, we utilized an improved technique for the order of preference by similarity to ideal solution (TOPSIS) to combine scores from distinct resources. This method was evaluated on two publicly available datasets regarding prostate cancer and lung cancer to identify disease-related genes. Consequently, our proposed strategy for gene prioritization showed its superiority to conventional methods in discovering significant disease-related genes with several types of genetic resources, while making good use of potential complementarities among available resources.

## 1. Introduction

The recent advance in high-throughput experiment technologies like microarrays and next-generation sequencing technologies has led to the production of large amounts of various biological resources regarding human genetic and disease-oriented data. Thus, it became one of the most significant issues in current biomedical research to identify disease genetic markers by exploring such a variety of resources in a systematic way. For this purpose, many earlier works [1–10] have been done by prioritizing candidate genes based on gene expression profiles or SNP genotype data, but they often produce many false-positive results, leading to the increase of time and cost to validate them experimentally.

In differential gene expression studies, the most common approach for gene prioritization is to utilize statistical methods for case-control microarray data which include *t*-test and significance analysis of microarrays (SAM) [1]. In these methods, candidate genes are prioritized according to *P* values and disease markers are chosen as such genes that have *P* values lower than a specific threshold. Other methods like fold change or information gain are also used to select probable disease-associated genes. On the other hand, genome-wide association studies (GWAS) are often made to identify genetic variations associated with specific diseases. For this purpose, some statistical methods, like the Cochran-Armitage trend test (CATT) [2, 3], the genotypic *χ* ^2^ test, and the allelic *χ* ^2^ test [4], are widely used. The CATT based on a specific genetic model usually performs better than Pearson's *χ* ^2^ test with 2 degrees of freedom [5] and has therefore been suggested for use in the analysis of case-control data [6]. Since the underlying genetic models of a complex disease are often unknown, the CATT is widely used in combination with an additive model. Although GWAS can identify SNPs and other variations in DNA that are associated with specific diseases, they cannot determine specific causal genes [7, 8]. In order to link the SNP-level data to the gene-level data, Lehne et al. [9] proposed the use of MaxT, MeanT, and TopQ since each gene might have several SNPs assigned to it.

There have also been extensive studies to examine expression quantitative trait loci (eQTL), which regulate mRNA and protein expression levels [10]. The eQTL can provide great insights into the molecular mechanisms underlying complex traits and aid in elucidating regulatory networks [11]. Furthermore, because eQTL data allow for the mapping of SNPs to biologically relevant genes [12], the Sherlock algorithm [13] employed the SNP and eQTL data to discover potential disease genes.

In spite of many positive aspects, however, these methods for gene prioritization have some disadvantages. For example, differential gene expression studies and GWAS focus only on a single data type for gene prioritization, so they suffer from being limited to a single genetic resource. This results that some potential disease-associated genes identified in differential gene expression studies remain undetected in GWAS, and vice versa. Also, both of them tend to show high false-positive rates. Thus, lately, an increasing number of gene prioritization tools are interested in integrating data from several resources [14–16].

In this paper, we propose a meta-analysis strategy for gene prioritization which integrates three different genetic resources, namely, gene expression data, SNP genotype data, and eQTL data, with an improved technique for order of preference by similarity to ideal solution (TOPSIS) [17]. The key idea of the proposed strategy is to utilize additional gene-level data obtained by using the eQTL data that provides SNP-gene mapping relationship and to combine the significance scores of candidate genes from three genetic resources with the improved TOPSIS. Our experiment results showed excellent performance of the proposed strategy in discovering significant disease-related genes.

## 2. Materials and Methods

### 2.1. Test Methods for Individual Genetic Resources

For the significance testing of gene expression data and SNP genotype data, we used the *t*-test and the CATT, respectively, which are the most commonly used ones in differential gene expression studies and GWAS, respectively.

### 2.2. Methods for Filtering Out Duplicate Gene Scores

*Max.* It is to choose the best score from duplicate gene scores for a certain gene. For example, in the case of *t*-test, SAM, and CATT analyses, the smallest *P* value is selected, and for information gain, the largest score is selected.

*TopQ.* It is to select the best quartile of all the duplicate scores given to a gene and use their arithmetic mean as a representative score of the gene. Here, if the number of the duplicate scores given to a gene is not a multiple of 4, the quartile number should be rounded up to the next integer.

*Mean.* It is to calculate the arithmetic mean of duplicate gene scores as a representative score of a certain gene.

### 2.3. Methods for Integrating Scores from Different Genetic Resources

*The Improved TOPSIS Method.* The original TOPSIS [18] is a method to measure comprehensive benefit of an object based on its relative distance to the ideal solution. The basic idea of this method is to find the positive ideal solution and the negative ideal solution in a decision-making process and then choose the alternatives in the descending order of the similarities to the positive ideal solution and in the ascending order of the distance to the negative ideal solution. On the other hand, the improved TOPSIS, which is a modified version of the original TOPSIS, was proposed as an evaluation method for economic problem [17]. To adapt this method for gene prioritization problem, we slightly modified the improved TOPSIS method. The detailed description of the procedure is given in the following.

(1) Firstly, construct a *n* × *m* gene score matrix where *n* is the total number of genes and *m* is the number of gene scores obtained from different genetic resources:
  (1) $$\begin{array}{c} X=\left[\begin{array}{cccc} X_{11} & X_{12} & \cdots & X_{1m}\\ X_{21} & X_{22} & \cdots & X_{2m}\\ \vdots & \vdots & \vdots & \vdots \\ X_{n1} & X_{n2} & \cdots & X_{nm} \end{array}\right]. \end{array}$$
 There can be some values missing in the matrix when the corresponding genes might not exist in the score list of some of the genetic resources. However, the improved TOPSIS method is not affected by missing values because it simply integrates existing scores only for specific genes.
(2) Secondly, normalize the gene scores in each individual resource by dividing each of them by the Euclidean norm of all the gene scores from the same resource, as in formula (2); for *i* = 1,2,…, *n* and *j* = 1,2,…, *m*,
  (2) $$\begin{array}{c} U_{ij}=\frac{X_{ij}}{\sqrt{\sum_{i=1}^{n}X_{ij}^{2}}}. \end{array}$$
(3) Thirdly, obtain the* most* positive solution (*U*_*j*_^+^) and the* most* negative solution (*U*_*j*_^−^) for each type of genetic resources. That is, for *i* = 1,2,…, *n* and *j* = 1,2,…, *m*, calculate the following:
  (3) $$\begin{array}{c} U^{+}=\left(U_{1}^{+},U_{2}^{+},\ldots ,U_{m}^{+}\right),\;U_{j}^{+}=\max\left\{U_{ij}\right\},\\ U^{-}=\left(U_{1}^{-},U_{2}^{-},\ldots ,U_{m}^{-}\right),\;U_{j}^{-}=\min\left\{U_{ij}\right\}. \end{array}$$
 Here the max indicates the selection of the most positive solution which is the best score chosen by taking the smallest *P* value from the *t*-test, SAM, or CATT results or taking the largest score from the results of information gain. On the other hand, the min indicates the selection of the most negative solution which is the worst score chosen by taking the largest *P* value for the *t*-test, SAM, or CATT results or taking the smallest score for the results of information gain.
(4) Finally, for each gene *i* = 1,2,…, *n*, calculate its relative distance *d*_*i*_ to the most negative solution by using formula (4), and then select the genes which have the larger values of *d*_*i*_ to find more significant genes:
  (4) $$\begin{array}{c} d_{i}=\frac{\langle \Delta U_{i},\Delta U\rangle }{{\left\|\Delta U\right\|}^{2}}, \end{array}$$
 where <, > indicates the inter product and ‖·‖ is the Euclidean norm. Consider
  (5) $$\begin{array}{c} \Delta U_{i}=\left(U_{i}-U^{-}\right),\\ \Delta U=\left(U^{+}-U^{-}\right),\\ \left\|\Delta U\right\|=\sqrt{\overset{m}{\underset{j=1}{\sum }}{\left(U_{j}^{+}-U_{j}^{-}\right)}^{2}}. \end{array}$$

*Rank Product (see [19]).* This method is to combine ranked lists for prioritization by using the following formula:

(6) $$\begin{array}{c} {\text{RP}}_{g}={\left(\overset{k}{\underset{i}{\prod }}r_{g,i}\right)}^{1/k}, \end{array}$$

where *r*_*g*,*i*_ is the rank of gene *g* in the score list of the *i*th genetic resource. That is, for each gene, it computes the rank product via the geometric mean of the ranks in the score lists of different genetic resources. Then the rank product is used as a final score for gene prioritization.

*Fisher's Method (see [20]).* This method combines extreme value probabilities from several tests, commonly known as *P* values, into one test statistic (*χ* ^2^) using the formula given in the following:

(7) $$\begin{array}{c} \chi_{2k}^{2}\sim -2\overset{k}{\underset{i=1}{\sum }}\ln\left(p_{i}\right). \end{array}$$

*Rescaled Sum of Z-Scores (see [21]).* This method combines several individual *Z*-scores by using the formula of

(8) $$\begin{array}{c} RSZ=\frac{\sum_{i=1}^{k}Z_{i}}{\sqrt{k}}, \end{array}$$

where *k* is the number of *Z*-scores to be combined.

## 3. Results and Discussion

### 3.1. Evaluation of Gene Prioritization Results Obtained by Integrating Genetic Resources with Improved TOPSIS

To evaluate the proposed strategy of gene prioritization integrating different genetic resources with the improved TOPSIS, we made experiments with two different datasets regarding prostate cancer and lung cancer. For each dataset, we downloaded gene expression data, SNP genotype data, and eQTL data from publicly available databases. In particular, for prostate cancer data, we used the gene expression profiles of the GSE6919 dataset [22] which includes 128 samples of 65 cases and 63 controls as in [23]. For SNP genotype data, we used the GSE18333 dataset [24] excluding 10 United Kingdom samples from original 82 samples, and this left us with 72 Chinese samples of 39 cases and 33 controls. For lung cancer data, we used the GSE19804 dataset [25], which includes 120 samples of 60 cases and 60 controls, as the gene expression data. For SNP genotype data, we used the GSE33355 dataset [26] of 122 samples with 61 cases and 61 controls. Also, Affymetrix 6.0 eQTL data were used which are downloadable from SCANdb [27]. Finally, for the validation of gene prioritization results, we downloaded the details of disease-associated genes from the Gene Association Database (GAD) and found 786 prostate cancer related genes and 731 lung cancer related genes, respectively.

**Figure 1:**
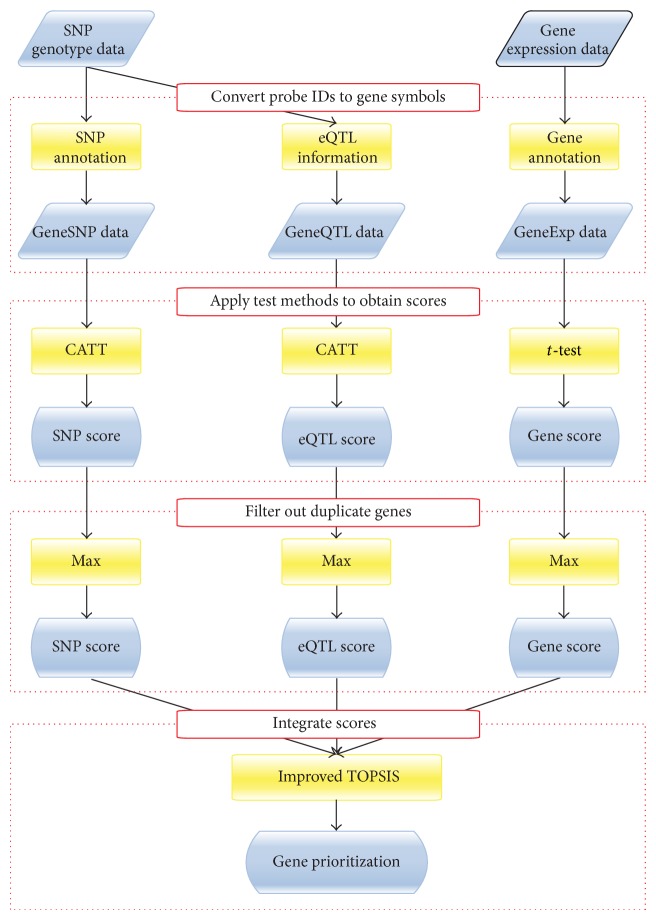
Overall procedure of the proposed strategy for gene prioritization which consists of four steps: (1) convert probe IDs to gene symbols, (2) apply test methods to obtain scores, (3) filter out duplicate genes in each score list, and (4) integrate scores with improved TOPSIS.

The overall procedure of the proposed strategy for gene prioritization is illustrated in Figure 1. To begin with, we preprocessed the gene expression data for specific disease by using the comprehensive robust multiarray average [28] method and produced the prostate cancer gene expression data consisting of 12,625 probes and 128 samples with 65 cases and 63 controls, and the lung cancer gene expression data consisted of 54,675 probes and 120 samples with 60 cases and 60 controls. For the processing of SNP genotype data, we removed such SNPs satisfying minimum allele frequency <0.01 and Hardy-Weinberg equilibrium test statistic value lower than ~7. Consequently, we obtained the prostate cancer SNP genotype data consisting of 709,216 SNPs and 72 samples with 39 cases and 33 controls, and the lung cancer SNP genotype data consisted of 760,716 SNPs and 122 samples with 61 cases and 61 controls. Next, with the above preprocessed data of gene expression and SNP genotype, we converted the probe IDs (or SNP IDs) to gene symbols with gene (or SNP) annotations, producing two datasets named GeneExp data and GeneSNP data, respectively. Also, by using eQTL data that conveys the biological relationships between SNPs and their regulated genes, we converted SNP IDs in the SNP genotype data to gene symbols, producing another dataset named GeneQTL data. Thus, eventually, it resulted in generating three datasets of genes (i.e., GeneExp data, GeneSNP data, and GeneQTL data), where each dataset may contain duplicate genes occurring by multiple probes mapped into the same gene symbol. These duplicate genes, if any, were filtered out after obtaining gene scores for each dataset.

**Figure 2:**
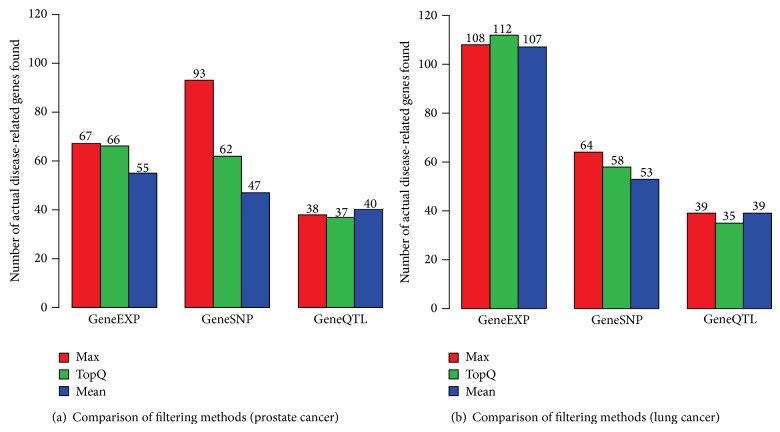
Comparison of the Max, TopQ, and Mean methods in filtering out duplicate genes: (a) prostate cancer results, (b) lung cancer results.

In order to obtain gene scores, we applied the most common test methods for the three datasets, respectively. The *t*-test was used for GeneExp data and the CATT was used for GeneSNP and GeneQTL data. Instead of these methods, any other common methods can be applicable for each dataset. Now, we have three datasets of gene scores from the GeneExp, GeneSNP, and GeneQTL data, which are named Gene scores, SNP scores, and eQTL scores, respectively. Based on these, we filtered out the duplicate genes by using one of the three available methods [9]: Max, TopQ, and Mean. In our experiments, we applied all three methods to remove duplicate genes and compared them in terms of the ability to discover potential disease-associated genes. Specifically, we chose the top 10% genes from each score list (ranked by *P* values) and, among them, counted the number of* actual* disease-related genes. The results from prostate cancer data and lung cancer data are shown in Figures 2(a) and 2(b), respectively, which clearly demonstrate that the Max method is the most suitable for filtering out duplicate genes. Thus, the Max method was used in all subsequent analyses. Consequently, after filtering out duplicate genes, we could obtain the prostate cancer dataset that includes 9,072 gene scores in the GeneExp data, 21,243 gene scores in the GeneSNP data, and 11,860 gene scores in the GeneQTL data. Similarly, for the lung cancer dataset, we obtained 22,635 gene scores in the GeneExp data, 21,393 gene scores in the GeneSNP data, and 11,860 genes scores in the GeneQTL data.

Finally, with these three kinds of gene scores, we applied the improved TOPSIS method to integrate them and prioritized candidate genes according to the combined score. It should be noted that the candidate genes here to be prioritized are as many as the union of the genes in GeneExp data, GeneSNP data, and GeneQTL data, which leads to the maximal use of distinct genetic resources.

**Figure 3:**
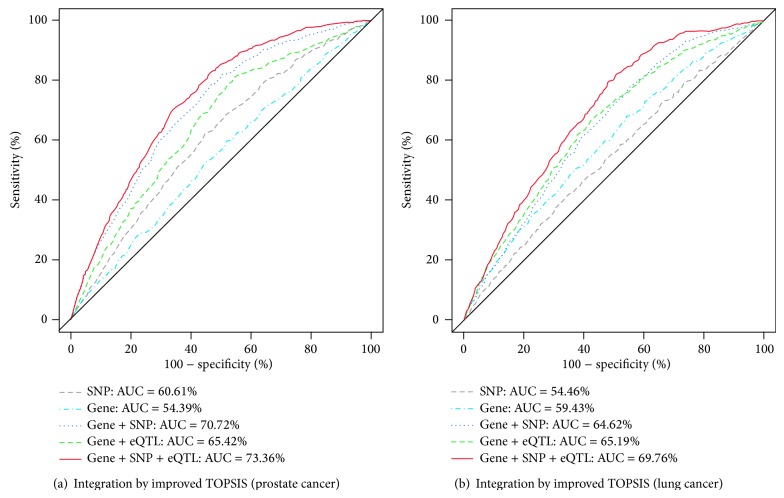
Effects of integrating distinct genetic resources with improved TOPSIS on disease-related gene identification: (a) prostate cancer results, (b) lung cancer results.

**Figure 4:**
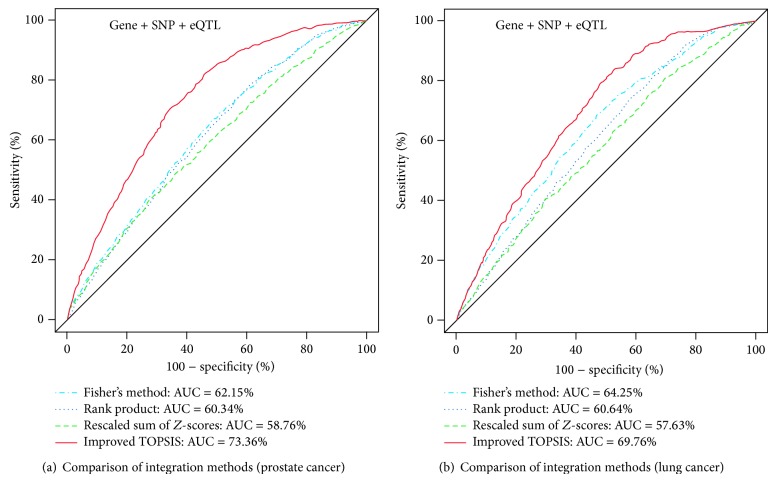
Comparison of the improved TOPSIS with other integration methods in terms of the ability to discover actual disease-related genes: (a) prostate cancer results, (b) lung cancer results.

Our experiment results of gene prioritization are summarized in Figures 2, 3, and 4, where the performance was evaluated in terms of the receiver operating characteristic (ROC) curves and the area under the curve (AUC) estimates. In particular, Figure 3 shows the effects of integrating distinct genetic resources with improved TOPSIS on disease-related gene identification in (a) prostate cancer data and (b) lung cancer data, respectively. From these figures, it is observed that the increasing number of distinct genetic resources to be used can be quite helpful to improve the performance of discovering potential disease-related genes, especially when the improved TOPSIS is used for the integration of different resources. In addition, this method does not only have the ability to cover as many genes as the union of the genes in different resources, but also can make good use of the potential complementarities among them.

### 3.2. Comparison of the Improved TOPSIS with Other Integration Methods

For the evaluation of our integrative approach with the improved TOPSIS, we also tested other integration methods (i.e., rank product, Fisher's method, and rescaled sum of *Z*-scores) under the same environment as in our experiments with the improved TOPSIS.  Figure 4 shows the comparison of the improved TOPSIS with other integration methods in terms of the ability to discover actual disease-related genes in (a) prostate cancer data and (b) lung cancer data. According to these results, the improved TOPSIS performed much better in integrating scores from three distinct genetic resources (Gene, SNP, eQTL data) than the other methods. This may be the reason that only the improved TOPSIS can provide higher ranks to the genes found in all the three genetic resources than those found in a single resource or any two resources, whereas the other methods cannot do so. From the formulas of the rank product, Fisher's method, and rescaled sum of *Z*-scores, which are introduced in the Methods, we can understand how such results can be obtained. For example, consider the case of two genes, in which the first gene's rank list is (1, 2, 3) and second gene's rank list is (3, NA, 1), where “NA” means that the gene is not present in the second genetic resource. When applying the rank product method to this case, the first gene's rank product is 1.82 and the second gene's rank product is 1.73. As a result, this method places the second gene in higher rank than the first gene, even though the first gene is actually much more important because it is present in all genetic resources. The Fisher and rescaled sum of *Z*-scores methods have similar problems. Consequently, it seems that such integration methods like the rank product method, Fisher's methods, and rescaled sum of *Z*-scores, are not suitable for integrating scores from these types of genetic resources.

### 3.3. Comparison of Our Strategy with Other Gene Prioritization Tools

**Figure 5:**
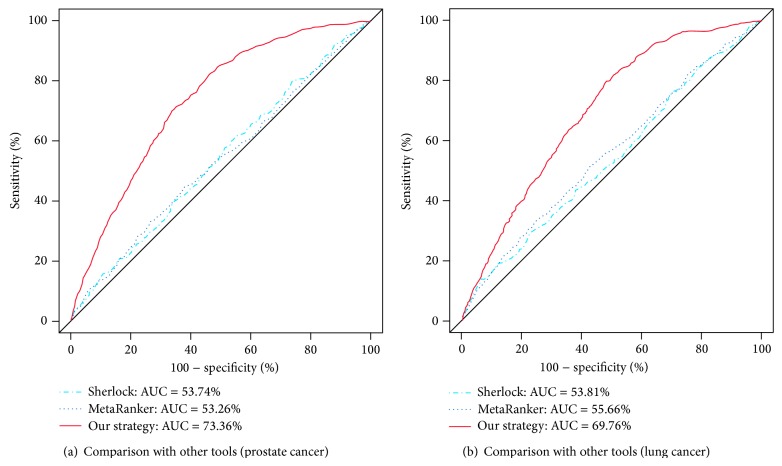
Comparison of our gene prioritization strategy with other meta-analysis tools in identifying actual disease-related genes: (a) prostate cancer results, (b) lung cancer results.

For comparative purpose, we performed similar experiments with two existing meta-analysis tools for gene prioritization, MetaRanker 2.0 and Sherlock. The MetaRanker 2.0 is a web-based gene prioritization tool in which several types of data from different genetic resources can be given as inputs. For our analyses with this tool, we used the same three genetic resources as in our earlier experiments, including SNP genotype data, gene expression data, and eQTL data. On the other hand, the Sherlock is a tool to discover disease-related genes via genome-wide association study using eQTL information. For experiments with the Sherlock, we used SNP genotype data (which is the same as in our earlier experiments) and the eQTL data used in [29] which is available to choose at the webpage of the Sherlock.  Figure 5 shows the comparison of our proposed strategy with these tools in identifying disease-related genes from (a) prostate cancer data and (b) lung cancer data. From these figures, it can be clearly observed that our integrative strategy for gene prioritization is superior to other meta-analysis tools, such as Sherlock and MetaRanker 2.0. Specifically, for prostate cancer data, our strategy showed 73.36% AUC estimate in identifying disease-related genes while the Sherlock and the MetaRanker 2.0 showed 53.74% and 53.26% AUC estimates respectively. Similarly, our strategy showed 69.76% AUC estimate in lung cancer related gene identification that has much better performance than the others, 53.81% AUC estimate in the Sherlock, and 55.66% AUC estimate in the MetaRanker 2.0.

## 4. Conclusions

In this paper we proposed an integrative strategy of gene prioritization which can employ various genetic resources, including gene expression data, SNP genotype data, and eQTL data, even if it is not limited to use these data only. Particularly, for the integration of scores from different resources, we used the improved TOPSIS method and could make good use of potential complementarities among available genetic resources. To verify the performance of our proposed strategy, we conducted experiments with two datasets regarding prostate cancer and lung cancer, each of which includes gene expression data, SNP genotype data, and eQTL data. The results demonstrate that our integrative strategy with the improved TOPSIS is superior to other integration methods in combining scores from distinct genetic resources, leading to the better performance in discovering disease-related genes. In addition, compared to other existing gene prioritization tools, our strategy is easily extensible and customizable to use many other resources for the meta-analysis, while producing very impressive results of gene prioritization.

To extend the present work, we are currently developing a web-based application to implement the proposed strategy. The first test version can be found at http://155.230.107.81/meta.analysis/.
